# Using established biorepositories for emerging research questions: a feasibility study

**DOI:** 10.1186/s12014-024-09504-6

**Published:** 2024-08-17

**Authors:** Lente J. S. Lerink, Christopher W. Sutton, Henny G. Otten, PROCARE consortium, Letizia Lo Faro, Rutger J. Ploeg, Jan H. N. Lindeman, Sadr Shaheed

**Affiliations:** 1 Nuffield Department of Surgical Sciences, University of Oxford, Oxford, UK; 2 Department of Surgery, Transplant Centre, Leiden University Medical Centre, Leiden, The Netherlands; 3 Institute of Cancer Therapeutics, University of Bradford, Bradford, UK; 4 Central Diagnostic Laboratory/Center for Translational Immunology, University Medical Centre Utrecht, Utrecht, The Netherlands; 5 NIHR Oxford Biomedical Research Centre, University of Oxford, Oxford, UK

**Keywords:** Kidney transplantation, Proteomics, Metabolomics, Biobank, Serum, Plasma

## Abstract

**Background:**

Proteomics and metabolomics offer substantial potential for advancing kidney transplant research by providing versatile opportunities for gaining insights into the biomolecular processes occurring in donors, recipients, and grafts. To achieve this, adequate quality and numbers of biological samples are required. Whilst access to donor samples is facilitated by initiatives such as the QUOD biobank, an adequately powered biobank allowing exploration of recipient-related aspects in long-term transplant outcomes is missing. Rich, yet unverified resources of recipient material are the serum repositories present in the immunological laboratories of kidney transplant centers that prospectively collect recipient sera for immunological monitoring. However, it is yet unsure whether these samples are also suitable for -omics applications, since such clinical samples are collected and stored by individual centers using non-uniform protocols and undergo an undocumented number of freeze–thaw cycles. Whilst these handling and storage aspects may affect individual proteins and metabolites, it was reasoned that incidental handling/storage artifacts will have a limited effect on a theoretical network (pathway) analysis. To test the potential of such long-term stored clinical serum samples for pathway profiling, we submitted these samples to discovery proteomics and metabolomics.

**Methods:**

A mass spectrometry-based shotgun discovery approach was used to obtain an overview of proteins and metabolites in clinical serum samples from the immunological laboratories of the Dutch PROCARE consortium. Parallel analyses were performed with material from the strictly protocolized QUOD biobank.

**Results:**

Following metabolomics, more than 800 compounds could be identified in both sample groups, of which 163 endogenous metabolites were found in samples from both biorepositories. Proteomics yielded more than 600 proteins in both groups. Despite the higher prevalence of fragments in the clinical, non-uniformly collected samples compared to the biobanked ones (42.5% vs 26.5% of their proteomes, respectively), these fragments could still be connected to their parent proteins. Next, the proteomic and metabolomic profiles were successfully mapped onto theoretical pathways through integrated pathway analysis, which showed significant enrichment of 79 pathways.

**Conclusions:**

This feasibility study demonstrated that long-term stored serum samples from clinical biorepositories can be used for qualitative proteomic and metabolomic pathway analysis, a notion with far-reaching implications for all biomedical, long-term outcome-dependent research questions and studies focusing on rare events.

**Supplementary Information:**

The online version contains supplementary material available at 10.1186/s12014-024-09504-6.

## Background

Proteomics and metabolomics are increasingly applied in kidney transplant research; in-detail exploration of molecules not only paves the way for e.g. biomarker development, but also greatly contributes to a better understanding of the relevant biomolecular processes in transplant donors, recipients, or grafts [1–4]. A critical factor to perform these analyses is the availability of adequate quality and numbers of biological samples, which is especially challenging when studying a large spectrum of outcomes of interest—including longer-term outcomes and more rare, but serious complications such as early graft loss or primary non-function.

In the past decade, the UK Quality in Organ Donation (QUOD) initiative has facilitated in-depth analysis of donor factors involved in transplant outcomes by allowing both access to a strictly protocolized sample collection as well as the accompanying epidemiological data [5]. Whilst QUOD provides ample samples suitable for -omics applications to explore the role of donor and procedural aspects on kidney transplant outcomes, an analogous and adequately powered initiative allowing exploration of the role of the recipient’s biomolecular aspects in transplant outcomes is missing. In the light of the increasing awareness that recipient factors may play a crucial part in kidney transplant outcomes [6, 7], the need for recipient-related resources to answer transplant-related questions is pressing. On the other hand, it takes years, if not decades, to establish a sufficiently powered biobanking initiative that enables the assessment of both long-term transplant outcomes and rare complications [8].

Rich and available, yet unverified resources of recipient material are the serum repositories present in the immunological surveillances labs that prospectively bank recipient sera for immunological monitoring. Aiming to study immunological determinants of kidney transplant outcomes, the Dutch immunological laboratories in transplant centres have established a national serum repository including sera from all recipients transplanted with a deceased donor graft between 1995 through 2005 [9]. Unlike the uniform and strict procedures now commonly applied by most biobanks, these long-term stored clinical samples were handled and stored according to different local protocols and were subjected to an unknown number of freeze–thaw cycles. While the utilization of this biorepository may hold significant implications for recipient-related research, it is widely recognized that proteins and metabolites can be influenced to varying degrees by handling and storage conditions [10–12], which may, in turn, affect the reliability of conclusions drawn for individual molecules. However, it is unclear to what extent the non-uniform acquisition and storage procedures interfere with explorative -omics/bioinformatics analysis based on wider biological networks, rather than individual molecules.

To assess whether (recipient) samples collected using clinical, local procedures, and stored for considerable periods of time can still be used for pathway profiling, we performed state-of-the-art proteomics and metabolomics [13]. A shotgun discovery approach was applied as this provides information on a broad variety of metabolites and proteins, and parallel analyses were performed with material from the QUOD biobank to check for any discrepancies. After obtaining adequate metabolomic and proteomic results for both types of samples, an integrated approach was applied to confirm pathway integrity. Results of this feasibility study provide insight in the value of using older, clinical bioliquids for multi-omics related research.

## Methods

### Sample collection

The extended storage, clinical sample group contained 30 serum samples from the Dutch PROCARE consortium, which was approved by the Research Ethics Committee for Biobanks and the Medical Ethics Committee of the University Medical Center Utrecht [9]. The selected samples originated from patients who received a kidney graft between 1996 and 2005 (Additional File 3: Table S1). Recipient serum samples were generally collected within three months prior to transplantation by various participating centres and handled using (non-uniform) local clinical protocols. The serum samples were decomplemented for 30 min at 37 °C, and subsequently stored at -80°C in a central biobank. During their storage, they have undergone an undocumented number of freeze–thaw cycles. The samples were compared to strictly biobanked donor EDTA plasma samples, obtained between 2013 and 2018 by the Quality in Organ Donation (QUOD) UK biobank (REC reference number 18/NW/0187) (Additional File 3: Table S1). The samples were drawn during donor management, after which they were immediately processed by participating centres by centrifugation at 13,000 rcf for 15 min. Next, samples were transported to the central QUOD biobank, aliquoted and stored at – 80 °C. The samples underwent a total of three freeze–thaw cycles.

### Metabolomics analysis

Before analysis, every frozen plasma and serum sample was thawed at 4 °C and 2 µL of pooled stable isotope standards mix (MSK-QC-KIT, Cambridge Isotope Laboratories Inc) was added to each sample as quality control for LC–MS metabolomics analysis. A total of 80 µL extraction buffer (acetonitrile/methanol/acetone, 8:1:1 ratio) mixture was added to 10 µL plasma and serum to precipitate proteins. After vertexing, the mixture was placed on ice for 30 min to further precipitate proteins and was subsequently centrifuged at 13,000 rcf for 20 min at 4 °C. Seventy-five µL of supernatant was transferred to a new tube and was split into two equal volumes for both positive and negative mode LC–MS. Metabolites were dried using a Thermo Scientific Savant DNA120 SpeedVac Concentrator and subsequently stored at -80°C until LC–MS analysis. Two pooled samples were prepared for quality control (QC) purposes, one for plasma and one for serum, in order to assess the reproducibility and reliability of the LC–MS analysis. These pooled samples underwent sample preparation and analysis as described above, and QC of the mixed samples was performed at the beginning, middle, and end of the analysis. Untargeted metabolomics was performed on a Dionex Ultimate 3000 HPLC System (Thermo Fisher Scientific, Massachusetts, USA) and Orbitrap Fusion instrument (Thermo Fisher Scientific, Massachusetts, USA). Methodological details can be found in Additional File 1: Supplementary Methods.

### Proteomics analysis

Protein quantification of plasma and serum samples was performed by Bradford assay. Similar to the metabolomics procedure, two pooled QC samples of plasma and serum were prepared and analysed intermittently to assess the variance observed in the data throughout the sample preparation, data acquisition and data pre-processing steps. Equal amounts of protein (350 µg) from QC, plasma and serum samples were treated with Top14 Abundant Protein Depletion Mini Spin Columns (A36370, Thermo Fisher Scientific, Massachusetts, USA) according to manufacturer’s instructions, to deplete top 14 plasma and serum most abundant proteins. The depleted samples were subsequently precipitated by 1:6 acetone precipitation overnight at – 20 °C. The precipitated samples were subsequently centrifuged for 15 min at 13,000 rcf at 4 °C, and the supernatant was removed. The resulting pellets were dissolved in 8 M urea and denatured with 50 mM dithiothreitol by incubation at 60°C for 10 min. Subsequently, samples were alkylated with 100 mM iodoacetamide in the dark at RT for 30 min. Urea concentration was diluted to 2 M by addition of 50 mM ammonium bicarbonate buffer. Next, trypsin digestion with mass spectrometry grade proteases (#90058, Thermo Fisher Scientific, Massachusetts, USA) was performed at 37˚C for 12 h. An end concentration of 1% formic acid was added to quench the enzymatic activity of trypsin, and a drop in pH was confirmed. 5 µL of labelled peptides (1:32 volume, #88320, Thermo-Fisher) was added to the pooled samples for proteomics QC. The resultant peptide mixture was desalted using Bond Elut C18 columns (#12102001, Agilent Technologies, California, USA) and dried using a Thermo Scientific Savant DNA120 SpeedVac Concentrator and stored at − 80°C until LC–MS analysis. Details about the shotgun LC–MS proteomics approach are given in the Additional File 1: Supplementary Methods.

### Statistical analysis

Descriptive statistics for all variables was conducted using SPSS Statistics for Windows, version 25.0 (SPSS Inc., Chicago, USA), and GraphPad Prism version 8.0 for Windows (GraphPad Software, San Diego, California, USA). The enrichment analysis of metabolites identified in both groups by untargeted metabolomics was performed using the web-based tool MetaboAnalyst version 5.0 [14]. For the chemical class analysis of metabolites, the probability of overrepresentation was calculated and corrected using the Hypergeometric test with the HyperScore function in MetaboAnalyst. Functional analysis of proteins was carried out using the STRING (Search Tool for the Retrieval of Interacting Genes/Proteins, version 11.5) webserver [15], and graphical presentations were created with R and GraphPad Prism.

## Results

To assess to what extent proteomic and metabolic signals and their evaluation are influenced by sample handling and long-term storage, we selected serum samples (n = 30) from the kidney recipient serum repository of the Dutch PROCARE Consortium that were stored for periods up to 26 years. These were compared with donor plasma samples (n = 30) from the strictly protocolized QUOD biobanking initiative.

### Metabolome profiling

**Fig. 1:**
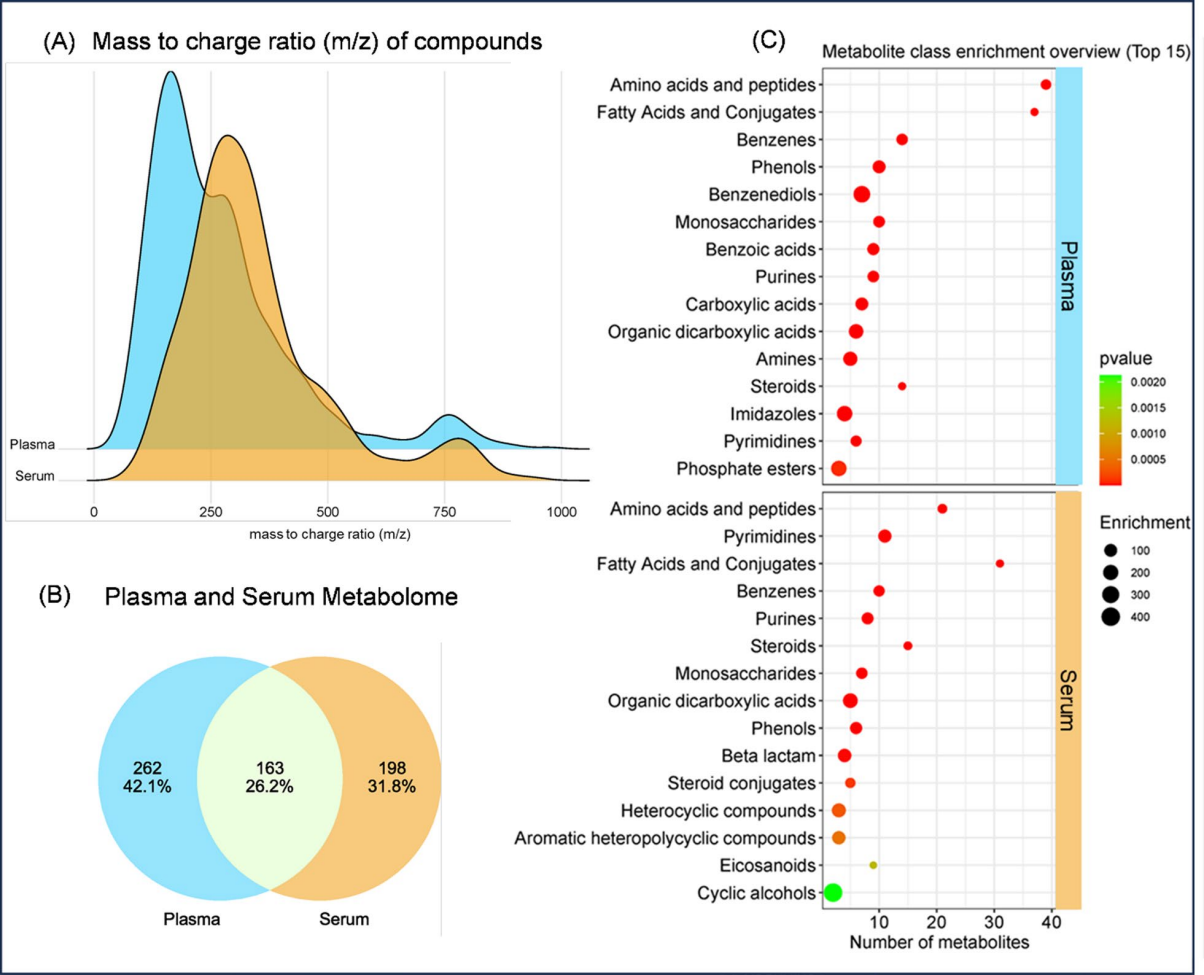
Global metabolome analysis of the plasma and serum samples. **A** Ridgeline plot presenting the distribution of mass to charge ratio (m/z) of compounds identified in plasma and serum samples. **B** Venn diagram highlighting the distribution of the identified endogenous metabolites per group in numbers and in percentage, evidencing the overlapping and unique metabolites. **C** Bubble plot of chemical class enrichment analysis of plasma and serum endogenous metabolites (top 15 sets, p < 0.05). The bubble size is correlated with the enrichment score, and a red colour means more significant enrichment. The X-axis depicts the number of metabolites identified in plasma and serum and the Y-axis lists the names of the chemical classes of annotated metabolites

Shotgun LC–MS metabolomics resulted in the identification of a total of 46,563 features when using 10 µL plasma (n = 30) and serum (n = 30) samples. Following manual validation and searching in mzCloud, KEGG and Human Metabolome databases, 803 and 891 compounds were identified in plasma and serum samples, respectively. The median mass to charge ratio (m/z) of identified compounds in plasma was 230 m/z, with an interquartile range (IQR) of 181–334 m/z, whilst for serum samples the median m/z was 313 (IQR 246–401 m/z) (Fig. 1A). The majority of the compounds (75%) identified in plasma and serum had a mass difference of < 2.0 parts per million between the observed mass of a compound and its annotated mass in the database, indicating high confidence data was acquired from both groups.

Subsequently, we especially aimed at exploring endogenous metabolites. A total of 623 endogenous metabolites were identified and quantified in both groups (Additional File 4: Table S2—Metabolome). Among those, 163 (26.2%) endogenous metabolites were present and common in the plasma and serum samples (Fig. 1B). For chemical classification of metabolites, we used the UCSD Metabolomics Workbench database [16] through the MetaboAnalyst platform. Both plasma and serum samples had significant (p < 0.05) recovery of “Amino acids and peptides”, “Fatty acids and conjugates”, “Benzene”, “Phenols”, “Monosaccharides”, “Organic dicarboxylic acids”, and “Steroids and conjugates” (Fig. 1C, Additional File 5: Table S3—Compounds class). When compared with the UCSD Metabolomics Workbench reference dataset, the “Amino acids and peptides” subset was the most common and enriched subset in both plasma (34.2-fold, p ~ 1.11E−46) and serum samples (23.3-fold, p ~ 3.09E−22).

### Proteome profiling

**Fig. 2:**
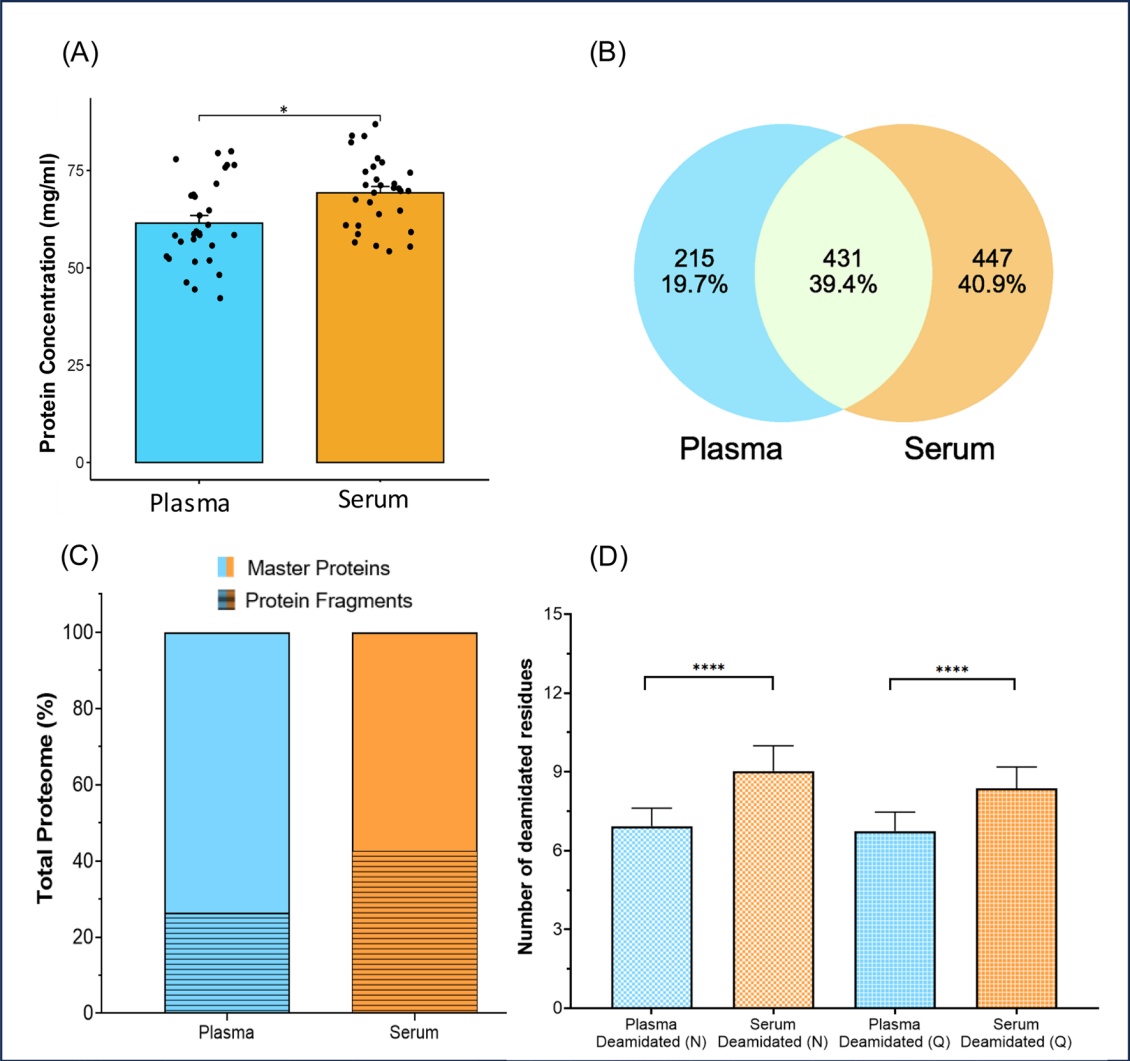
Global proteome analysis of the plasma and serum samples. **A** Venn diagram describing common and unique proteins between the plasma and serum samples. **B** Protein fragments level reported as percentage of total proteome. **C** Deamidation level of asparagine (N) and glutamine (Q) amino acids. **D** Oxidation level of Methionine (M) residue in the plasma and serum samples. “ns”; non-significant. (****); statistical significance p ≤ 0.0001

**Fig. 3:**
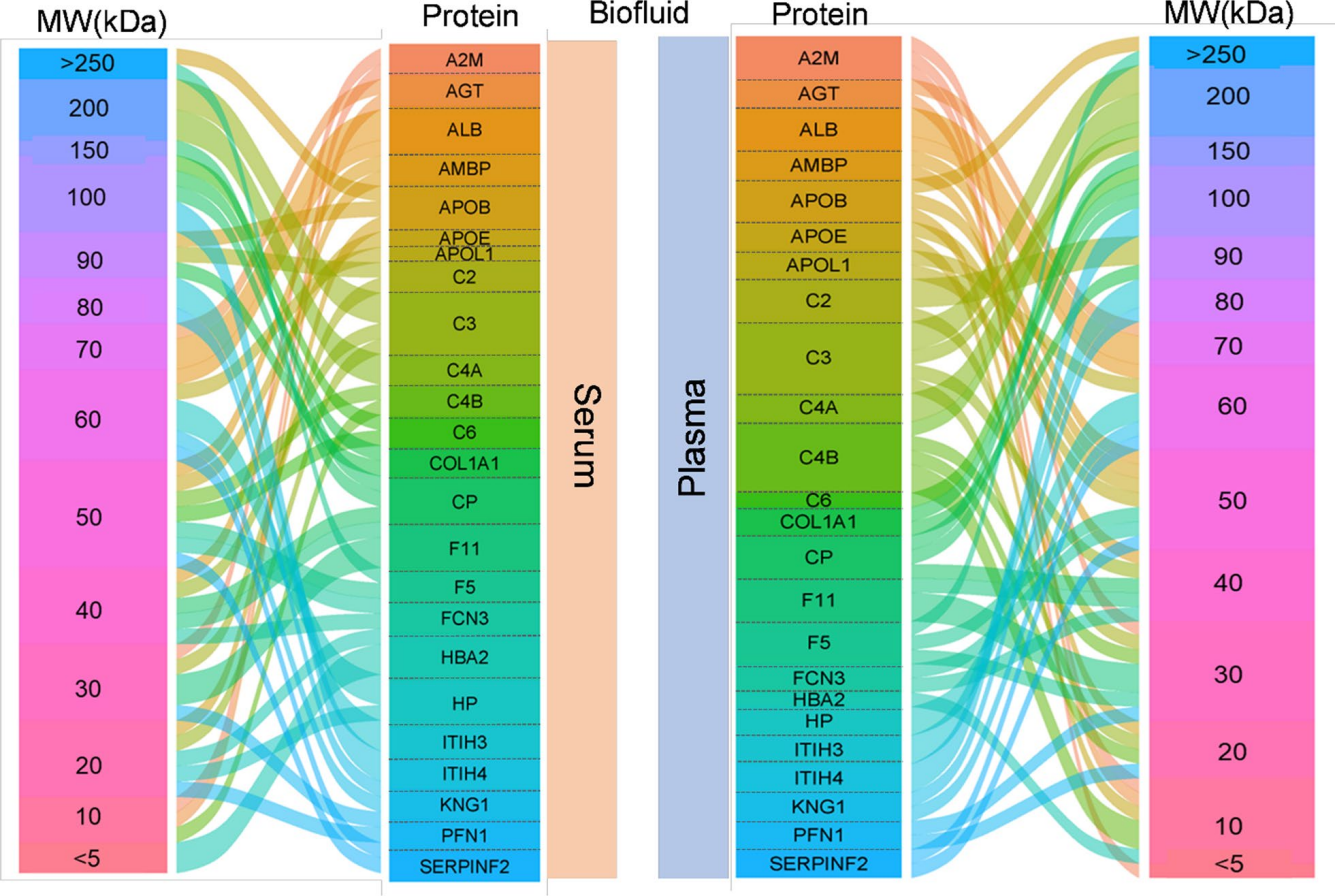
Visual representation of protein fragments through alluvial plots. The diagram flow shows the multitude of protein fragments with master protein and molecular weight (MW) which were identified in plasma and serum samples. The size of the coloured blocks in the protein column are proportional to the number of fragments identified for that specific master protein. The connecting lines between the protein and MW columns represent the identified fragments. The “MW” column provides information on the MW of the specified fragment, in kDa

The protein concentration of the plasma samples was 61.48 ± 10.48 mg/mL with a coefficient of variation (CV) of 17.59%. The protein concentration of the serum samples was higher: 69.28 ± 9.01 mg/mL with a CV of 13.01% (Additional File 2: Figure S1). Analysis of plasma and serum samples by shotgun proteomics identified a total of 1524 proteins (Additional File 6: Table S4—Proteome). In the donor plasma samples, a total of 6780 peptides were detected by LC–MS, which enabled the identification of 646 plasma proteins. In the recipient serum samples, 7777 peptides were identified, which resulted in the identification of 878 proteins. The molecular weight (MW) of plasma and serum sample proteins ranged from 2.2 to 515kDa, with a median of 44kDa (IQR 24–71kDa) for plasma and 29kDa (IQR 13–8kDa) for serum samples. A total of 431 (39.4%) of the identified proteins overlapped between the two groups (Fig. 2A). Since the serum and plasma groups differ biologically (recipient vs donor), we focused on the common proteins, i.e. proteins that were present in both groups. Further analysis of the common proteins demonstrated that in plasma samples, on average 12.2 peptides were identified per protein, and 13.7 peptides per protein for serum samples. The average sequence percentage of protein was 30.3% for plasma and 33.8% for serum. Compared to the plasma samples, relatively more proteins were identified in serum samples in the smaller mass range of 10-25kDa (Additional File 2: Figure S2). On analysis of these low molecular weight proteins in Uniprot database [17], 373 protein fragments (i.e. short segments of the peptide backbone) were identified in the serum samples proteome, compared to 171 protein fragments in the plasma samples (Fig. 2B), respectively covering 42.5% and 26.5% of their total proteomes. The identified fragments and their master proteins are visualized in Fig. 3.

The oxidation of methionine (M) and deamidation of asparagine (N) and glutamine (Q) are common chemical modifications that can occur in proteins during long-term storage of plasma and serum samples. These modifications can affect the integrity of the proteins and may impact the accuracy of determining their role in biological processes [18]. A total of 281 common proteins were identified with oxidation and deamidation in both biofluids. The rate of deamidation of asparagine and glutamine was significantly higher (p < 0.0001) in long-term stored serum samples compared to plasma group (Fig. 2C). No significant difference was observed in the oxidation of methionine residue in donor plasma and recipient serum (Fig. 2D).

### Integrated proteomics and metabolomics pathway analysis

**Fig. 4:**
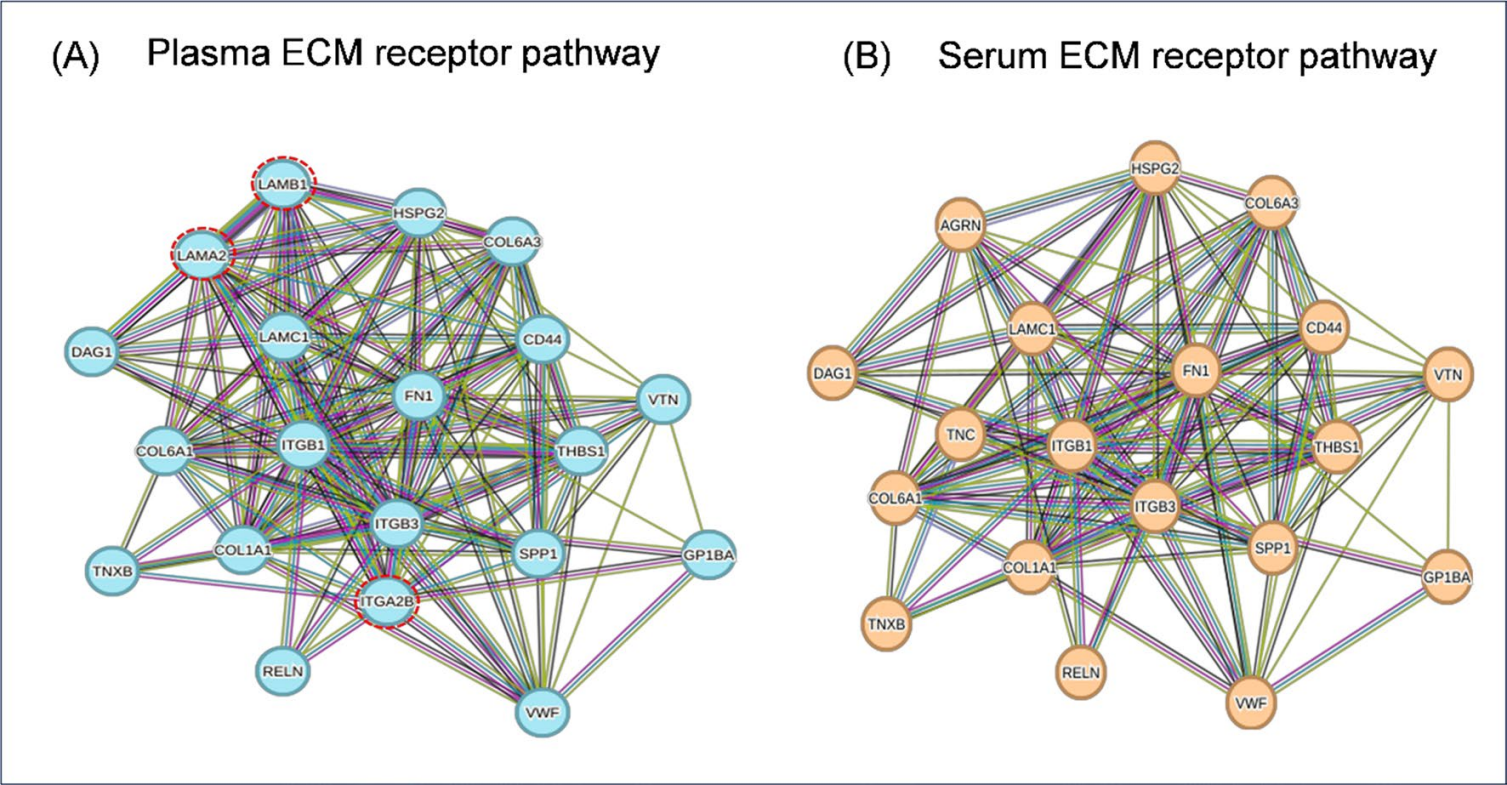
STRING analysis of the extracellular matrix (ECM) receptor interaction pathway. The network displays the interaction between the identified proteins in plasma (**A**) and serum (**B**) samples. Each node denotes a gene. The nodes circled with red indicate the genes uniquely identified in the plasma samples. The connecting lines between nodes indicate the evidence of their relationships: red line = fusion evidence; green line = neighbourhood evidence; blue line = co-occurrence evidence; purple line = experimental evidence; yellow line = text-mining evidence; light blue line = database evidence; black line = co-expression evidence

The above data show that it is technically possible to perform metabolomic and proteomic profiling of serum samples obtained 26 years ago under the PROCARE consortium. Yet, it is impossible to draw conclusions on individual metabolites or proteins due to the different natures of biofluids, the impact of storage and biological differences between (deceased) donors and listed recipients suffering of kidney failure. However, by virtue of its reliance on theoretical networks, integrated pathway enrichment analysis will be less affected by global artifacts in the proteome/metabolome related to storage or the difference between serum and plasma. Thus, to test whether these signatures might yield biologically relevant information, we performed integrated pathway enrichment analysis of proteins and metabolites using the MetaboAnalyst platform and KEGG pathway database. The integrated analysis of proteins and metabolites revealed that 79 pathways were significantly enriched (p < 0.05, Additional File 7: Table S5—Enriched Pathways) in plasma and serum samples compared to the KEGG reference database. The impact values of the majority (56%) of these pathways were greater than 0.2, indicating that this altered pathway is evident in the tested samples, and can thus be profiled. The extracellular matrix (ECM)-receptor interaction was one the most enriched pathway in both groups (plasma, p ~ 3.62E–11 and serum, p ~ 2.23E–11). These interactions play an important role in many cellular activities such as differentiation, proliferation, apoptosis, adhesion and migration, angiogenesis, and immune response and thus makes biological sense to be enriched in the sample groups [19]. A total of 20 members of ECM-receptor interaction pathway were identified in the plasma samples, of which 17 proteins were also present in the serum samples. Therefore, the large majority of the network had remained intact in the serum samples (Fig. 4). Only three members of ECM-receptor interaction pathway, integrin alpha-IIb (ITA2B), laminin subunit alpha-2 (LAMA2) and laminin subunit beta-1 (LAMB1), were not detected in the serum samples (Fig. 4).

## Discussion

Large, prospective biorepositories are a prerequisite in order to explore the molecular basis and biomarkers of favourable and unfavourable transplant outcomes. In the first large-scale transplant biobank, the UK QUOD initiative, donor samples and data are handled and stored according to a strict and uniform protocol and thereby facilitate donor-based, outcome-focused research. Unfortunately, since a similar initiative is not yet available for recipient samples, critical questions regarding a possible role of (molecular) recipient factors in transplant outcomes can currently not be adequately investigated nor addressed [6, 7]. Establishment of an adequately sized biobank that incorporates sufficient cases to address short term complications and has adequate long-term follow up information will be extremely time-consuming, thereby interfering with a timely evaluation of the role of recipient factors.

A possible alternative and rich source of recipient samples are the sera that have been stored for immunological matching and surveillance. However, since these sera have been collected by different centres using local clinical protocols, there probably is a large variability in sample handling and storage practices, whilst the material may also have been exposed to an unknown number of freeze–thaw cycles; factors that may all impact serum/plasma quality for future proteomic and metabolomic analysis [10–12]. In the light of the huge potential of these clinical sera, and the fact that a prospective collection will take years to gain any relevant numbers, we decided to investigate to what extent these samples might still provide relevant biological information to build biological pathways from. In this feasibility study, we applied a state-of-the-art shotgun and data-dependent acquisition metabolomics and proteomics approach in order to estimate the informative value of long-term stored, immunological surveillance lab-derived recipient serum samples, taking along uniformly biobanked QUOD donor plasma samples in parallel analyses.

A potential interference in this study is that it relies on two different sample groups. In an ideal world, one would have used uniform biological samples (serum or plasma) from a homogenous population (recipients or donors), and in parallel test fresh and long-term stored (> 20 years) samples. However, this is not feasible, and unfortunately, even a potential use of artificially aged samples as a reference, i.e., through exposing fresh samples to multiple freeze–thaw cycles, cannot reflect actual storage [10]. Nevertheless, the impact of non-uniform biological samples appears less than commonly thought, since serum and plasma proteomes are reportedly very similar, apart from fibrinogen levels [20]. To minimize the impact of biological differences, the pathway enrichments were performed against a reference database rather than one-to-one (i.e., plasma vs serum).

The advantage of the applied shotgun method is that it yields a broad overview of all proteins and metabolites present and is therefore an appropriate strategy for an explorative approach. However, it does not provide the optimal analytical circumstances for every specific compound, in contrast to a targeted approach. This is reflected e.g., by the incomplete identification of the amino acids class of components in both plasma and serum (Additional File 4 and 5). Also, for proteins, incomplete patterns were observed: i.e., complement protein C3 has nine protein fragments [17], while shotgun proteomics only identified three fragments in serum and four in plasma (Fig. 3). Nevertheless, the shotgun approach enables the generation of global metabolomics and proteomics data as required for pathway analysis, which is essential to answer biological questions.

In this feasibility study, LC–MS metabolomics was successfully applied in both groups: 891 and 803 compounds were identified in the recipient serum and donor plasma samples, respectively, of which 361 and 425 were endogenous metabolites. The majority (75%) of the compounds were identified with high confidence, and metabolites from similar metabolite classes were found in both groups (Fig. 1C), as expected for human blood samples. The median m/z ratio was higher for serum samples (313 m/z) compared to the plasma samples (230 m/z). Although a storage effect cannot be excluded, this difference more likely results from the clinical difference between the groups: whilst the plasma samples are derived from donors with adequate renal function, the serum samples all originate from patients suffering from end-stage renal disease, a condition known to result in accumulation of metabolites [21, 22], which could have contributed to the higher m/z ratio in the serum samples.

Although the measured protein concentrations were within the theoretical serum/plasma protein concentration range of 60–80 mg/mL [23], the concentration was higher in serum (69 ± 9 mg/mL) compared to plasma (61 ± 11 mg/mL), whilst the opposite was expected [24]. The most likely explanation is the prolonged storage of the serum samples, during which water will have evaporated, which has resulted in concentration of the sample. Whereas this affects absolute metabolite and protein abundance, and thus affects specific biomarker analysis, it does not impact the relative concentrations, and will therefore not interfere with general pathway analysis based on the presence of proteins rather than absolute abundances.

Using the power of shotgun LC–MS proteomics, it was possible to quantify not only the master proteins but also protein fragments in plasma and serum. In recipient serum, 42.5% of all proteins were fragments, whilst in the donor plasma group 26.5% of the proteins were fragments. This was also reflected in the size distribution with an overall lower MW of serum sample proteins compared to plasma. Although it cannot be ruled out that this increased fragmentation in kidney recipient sera is of biological origin [25], it is also an expected consequence of the non-uniform sampling and extended storage, which could e.g. lead to deamidation. In line with this high abundance of protein fragments in the serum samples of our study, a high level of deamidation (70%) was observed. Deamidation is a non-enzymatic, chemical post-translational modification that can occur in peptides and proteins during their lifespan (in vivo and in stored samples), which can have significant implications for the stability and integrity of these molecules in biological samples as it can make proteins more susceptible to degradation by proteolytic enzymes [26]. Whilst the observed fragmentation of proteins will interfere with analyses based on antibody-based assays or aptamer platforms, it has a limited effect on LC–MS-based pathway analysis; a fragment will still be identified as a fragment originating from its parent protein. The fragment can thus be taken along in the analysis as if it was an intact protein, if required.

The analytical strategy adequately generated metabolic and proteomic profiles from the recipient serum samples. To test to what extent the acquired data could be mapped along theoretical pathways, an integrated pathway analysis was performed in which enriched pathways in the recipient serum and donor plasma were mapped against the KEGG reference database. Integration of the qualitative proteomics and metabolomics data of both recipient serum and donor plasma samples resulted in the significant enrichment of a total of 79 pathways. Although the complement and coagulation cascades pathway had the highest impact and lowest p-value, interpretation of this pathway is potentially interfered as coagulation cascade activation in serum can trigger the complement cascade [27]. Consequently, this pathway is suboptimal for exploration of the potential impact of storage artifacts. Therefore, it was decided to focus on ECM-receptor interaction pathway as a universally enriched pathway for integrated pathway analysis. In-depth STRING analysis of this pathway showed major overlap between donor plasma and recipient serum samples (Fig. 4). Interestingly, with only three nodes missing from the serum analysis, the overall overview of the ECM receptor interaction pathway remained intact. This illustrates that older serum samples from non-uniform, clinical biorepositories can be applied for general qualitative proteomic and metabolomic integrated pathway analysis, yielding a resolution similar to that of strictly biobanked samples.

Besides the difference in biofluids, another limitation that especially affects metabolite abundances is the difference in nutritional state: the donor plasma samples are obtained from individuals who are in the process of dying and thus in a fasting, glucose-infusion state, whilst the serum samples originate from non-fasting recipients using a variety in diets. Moreover, donor type (brain death vs circulatory death) may also impact metabolism [28]. Again, we have therefore decided to not compare the two groups one-to-one.

## Conclusions

This feasibility study demonstrated that recipient serum samples that were obtained using local procedures, stored for long periods of time and subjected to an unknown number of freeze–thaw cycles can still be used for qualitative proteomic and metabolomic pathway analysis, yielding intact biological pathways. Using samples from pre-existing collections will enable us to tackle pressing molecular questions regarding long-term outcomes and rare events in the field of transplantation, as well as in any other biomedical research field dependent on large biorepositories. Whilst samples from uniformly protocolized biobanks obviously remain the gold standard, this study shows that samples from clinical biorepositories still provide useful information when applying integrated pathway analysis.

### Supplementary Information

Additional file 1.

Additional file 2.

Additional file 3.

Additional file 4.

Additional file 5.

Additional file 6.

Additional file 7.

## Data Availability

Raw files from the proteomic analysis along with all metadata were deposited in PRIDE with the project accession number PXD040856. Raw files of the metabolomic metadata are uploaded to MetaboLights number MTBLS8189.

## Abbreviations

CV: Coefficient of variation
ECM: Extracellular matrix
ITA2B: Integrin alpha-IIb
IQR: Interquartile range
LAMA2: Laminin subunit alpha-2
LAMB1: Laminin subunit beta-1
m/z: Mass to charge ratio
MW: Molecular weight

## References

[1] Stanimirova I, Banasik M, Ząbek A, Dawiskiba T, Kościelska-Kasprzak K, Wojtowicz W, et al. Serum metabolomics approach to monitor the changes in metabolite profiles following renal transplantation. Sci Rep. 2020;10(1):17223. 33057167 10.1038/s41598-020-74245-z PMC7560840

[2] Kim SC, Page EK, Knechtle SJ. Urine proteomics in kidney transplantation. Transplant Rev (Orlando). 2014;28(1):15–20. 24321302 10.1016/j.trre.2013.10.004

[3] Colas L, Royer AL, Massias J, Raux A, Chesneau M, Kerleau C, et al. Urinary metabolomic profiling from spontaneous tolerant kidney transplanted recipients shows enrichment in tryptophan-derived metabolites. EBioMedicine. 2022;77: 103844. 35241402 10.1016/j.ebiom.2022.103844 PMC9034456

[4] van Leeuwen LL, Spraakman NA, Brat A, Huang H, Thorne AM, Bonham S, et al. Proteomic analysis of machine perfusion solution from brain dead donor kidneys reveals that elevated complement, cytoskeleton and lipid metabolism proteins are associated with 1-year outcome. Transpl Int. 2021;34(9):1618–29. 34448265 10.1111/tri.13984 PMC9292651

[5] QUOD. https://www.nds.ox.ac.uk/research/quod.

[6] De Kok MJ, Schaapherder AF, Mensink JW, de Vries AP, Reinders ME, Konijn C, et al. A nationwide evaluation of deceased donor kidney transplantation indicates detrimental consequences of early graft loss. Kidney Int. 2020;97(6):1243–52. 32359810 10.1016/j.kint.2020.01.043

[7] Schaapherder AF, Kaisar M, Mumford L, Robb M, Johnson R, de Kok MJ, et al. Donor characteristics and their impact on kidney transplantation outcomes: results from two nationwide instrumental variable analyses based on outcomes of donor kidney pairs accepted for transplantation. EClinicalMedicine. 2022. 10.1016/j.eclinm.2022.101516. 35784435 10.1016/j.eclinm.2022.101516 PMC9240982

[8] Coppola L, Cianflone A, Grimaldi AM, Incoronato M, Bevilacqua P, Messina F, et al. Biobanking in health care: evolution and future directions. J Transl Med. 2019;17(1):172. 31118074 10.1186/s12967-019-1922-3 PMC6532145

[9] Kamburova E, Wisse B, Joosten I, Allebes W, Van Der Meer A, Hilbrands L, et al. Differential effects of donor-specific HLA antibodies in living versus deceased donor transplant. Am J Transplant. 2018;18(9):2274–84. 29464832 10.1111/ajt.14709 PMC6175247

[10] Mitchell BL, Yasui Y, Li CI, Fitzpatrick AL, Lampe PD. Impact of freeze-thaw cycles and storage time on plasma samples used in mass spectrometry based biomarker discovery projects. Cancer informatics. 2005;1:117693510500100110. 10.1177/117693510500100110 PMC2657648 19305635

[11] Wagner-Golbs A, Neuber S, Kamlage B, Christiansen N, Bethan B, Rennefahrt U, et al. Effects of long-term storage at− 80 C on the human plasma metabolome. Metabolites. 2019;9(5):99. 31108909 10.3390/metabo9050099 PMC6572224

[12] Enroth S, Hallmans G, Grankvist K, Gyllensten U. Effects of long-term storage time and original sampling month on biobank plasma protein concentrations. EBioMedicine. 2016;12:309–14. 27596149 10.1016/j.ebiom.2016.08.038 PMC5078583

[13] Zhang Y, Fonslow BR, Shan B, Baek MC, Yates JR 3rd. Protein analysis by shotgun/bottom-up proteomics. Chem Rev. 2013;113(4):2343–94. 10.1021/cr3003533. 23438204 10.1021/cr3003533 PMC3751594

[14] Pang Z, Chong J, Zhou G, de Lima Morais DA, Chang L, Barrette M, et al. MetaboAnalyst 5.0: narrowing the gap between raw spectra and functional insights. Nucleic Acids Res. 2021;49(W1):W388–96. 34019663 10.1093/nar/gkab382 PMC8265181

[15] Szklarczyk D, Gable AL, Nastou KC, Lyon D, Kirsch R, Pyysalo S, et al. The STRING database in 2021: customizable protein-protein networks, and functional characterization of user-uploaded gene/measurement sets. Nucleic Acids Res. 2021;49(D1):D605–12. 33237311 10.1093/nar/gkaa1074 PMC7779004

[16] Sud M, Fahy E, Cotter D, Azam K, Vadivelu I, Burant C, et al. Metabolomics workbench: an international repository for metabolomics data and metadata, metabolite standards, protocols, tutorials and training, and analysis tools. Nucleic Acids Res. 2016;44(D1):D463–70. 26467476 10.1093/nar/gkv1042 PMC4702780

[17] UniProt: the Universal Protein Knowledgebase in 2023. Nucleic Acids Res. 2023;51(D1):D523–31. 10.1093/nar/gkac1052. 10.1093/nar/gkac1052 PMC9825514 36408920

[18] Ying Y, Li H. Recent progress in the analysis of protein deamidation using mass spectrometry. Methods. 2022;200:42–57. 32544593 10.1016/j.ymeth.2020.06.009

[19] Karamanos NK, Theocharis AD, Piperigkou Z, Manou D, Passi A, Skandalis SS, et al. A guide to the composition and functions of the extracellular matrix. FEBS J. 2021;288(24):6850–912. 33605520 10.1111/febs.15776

[20] Zimmerman LJ, Li M, Yarbrough WG, Slebos RJ, Liebler DC. Global stability of plasma proteomes for mass spectrometry-based analyses. Mol Cell Proteom. 2012. 10.1074/mcp.M111.014340. 10.1074/mcp.M111.014340 PMC3433892 22301387

[21] Rhee EP, editor. A systems-level view of renal metabolomics. Seminars in nephrology; 2018: Elsevier. 10.1016/j.semnephrol.2018.01.005 PMC5880322 29602397

[22] Sirich TL, Funk BA, Plummer NS, Hostetter TH, Meyer TW. Prominent accumulation in hemodialysis patients of solutes normally cleared by tubular secretion. J Am Soc Nephrol. 2014;25(3):615. 24231664 10.1681/ASN.2013060597 PMC3935591

[23] Barrett KE. Ganong’s review of medical physiology2010.

[24] Lum G, Gambino SR. A comparison of serum versus heparinized plasma for routine chemistry tests. Am J Clin Pathol. 1974;61(1):108–13. 4809144 10.1093/ajcp/61.1.108

[25] Ramírez Medina CR, Ali I, Baricevic-Jones I, Odudu A, Saleem MA, Whetton AD, et al. Proteomic signature associated with chronic kidney disease (CKD) progression identified by data-independent acquisition mass spectrometry. Clin Proteom. 2023;20(1):1–12. 10.1186/s12014-023-09405-0 PMC10116780 37076799

[26] Hao P, Adav SS, Gallart-Palau X, Sze SK. Recent advances in mass spectrometric analysis of protein deamidation. Mass Spectrom Rev. 2017;36(6):677–92. 26763661 10.1002/mas.21491

[27] Amara U, Rittirsch D, Flierl M, Bruckner U, Klos A, Gebhard F, et al. Interaction between the coagulation and complement system. Curr Top Complement. 2008;II:68–76. 10.1007/978-0-387-78952-1_6 PMC2713875 19025115

[28] Dawiskiba T, Wojtowicz W, Qasem B, Łukaszewski M, Mielko KA, Dawiskiba A, et al. Brain-dead and coma patients exhibit different serum metabolic profiles: preliminary investigation of a novel diagnostic approach in neurocritical care. Sci Rep. 2021;11(1):15519. 34330941 10.1038/s41598-021-94625-3 PMC8324823

