# Supplementary material for: Using established biorepositories for emerging research questions: a feasibility study
Source: Clin Proteomics. 2024 Aug 17;21:54. doi: 10.1186/s12014-024-09504-6 (PMC11330044; doi:10.1186/s12014-024-09504-6)
Supplement: Supplementary file 1 — Additional file 1. [file 12014_2024_9504_MOESM1_ESM.docx]

Additional File 1 – Supplementary Methods

Applying established serum repositories for emerging research questions: a feasibility study.

1. **Shotgun LC-MS metabolomics**

Chromatographic separation was performed on a Dionex Ultimate 3000 HPLC System (Thermo Fisher Scientific, Massachusetts, USA) with a Hypersil Gold C18 column (150 × 2.1 mm, 1.9 µm; Thermo Fisher Scientific) under gradient conditions, using mobile phases A (0.1% formic acid in 2% acetonitrile: water, v/v) and B (0.1% formic acid in 90% acetonitrile: water, v/v). Flow rate was set at 350 µL/min and the column was maintained at 45°C. Extracted metabolites were dissolved in 20 µL solvent A and 5 µL of sample was injected into the column for each run. The gradient started with 99% A and decreased to 10% A in 17 min, holding at 10% A for 4 min, and then turned to 99% A immediately, holding at 99% A for 5 min. Mass spectrometry analyses were performed on an Orbitrap Fusion instrument (Thermo Fisher Scientific, Massachusetts, USA) fitted with a Heated Electrospray Ionization (HESI) source. Data were acquired in positive and negative modes for each LC-MS analysis. Source parameters for positive ionization mode were set as follows: spray voltage at +4.2 kV, sheath gas at 35 (arbitrary units), auxiliary gas at 10 (arbitrary units) and sweep gas at 2 (arbitrary units), while for negative ionization mode spray voltage - 3.2 kV, sheath gas 40, auxiliary gas 15 and sweep gas 1. The Ion transfer tube temperature was set at 300°C, and vaporizer temperature at 300°C. For MS1 acquisition, the data was acquired using the Orbitrap analyzer, at a resolution of 120,000 at m/z 200 (FWHM, a spectrum collected at this resolution requires ~0.6 s); maximum injection time was set at 250 ms, automatic gain control (AGC) target at 5.0 x 104, RF lens level at 60% and the scan range from m/z 70-1000. The MS2 acquisition was performed in Orbitrap with resolution was 30,000 at m/z 200, isolation window of 1.0, scan range “normal”, maximum injection time 35 ms. The selected ions were sequentially fragmented by higher energy collisional dissociation (HCD) with stepped collision energy of 10%, 30% and 50%. In all cases, one microscan was recorded using dynamic exclusion of 3 seconds and mass tolerance of ±5ppm. A targeted exclusion list of background noise with mass tolerance of ±5ppm was also included in the method to remove the LC-MS contaminants.

Raw LC-MS metabolomics data was processed using Compound Discoverer v.3.3.1.111 (Thermo Fisher Scientific, Massachusetts, USA), using mzCloud, Kyoto Encyclopedia of Genes and Genomes (KEGG) and Human Metabolome database. All these databases were accessed or downloaded on January 10, 2023. Compound Discoverer (CD) parameters were set as follows: MS1 tolerance, 5ppm; minimum peak height, 1,000; preferred ions, [M+H]+1; [M-H]-1; maximum retention time shift, 0.2 min; MS2 mass tolerance, 10ppm (for mzCloud library search). Fill gaps tool in CD was used to correct for possible peak detection or alignment errors for missing peaks and Systematic Error Removal with Random Forest (SERRF) correction beta was applied to remove the time-dependent batch effects, using pooled quality control (QC) samples. Finally, parameters for predict composition tool were adapted to analysis with MS1 and MS2 tolerance 5ppm, maximum element counts of C90 H190 Br3 Cl4 N10 O18 P3 S5 and signal to noise threshold of 3, in order to predict the chemical formulas of the unknown compounds. Background compounds were removed if the sample to blank area ratio was less than 5. Features identified with mass difference >5ppm between the observed mass of a compound and its annotated mass, and compounds with “not the top hit” or “partial match” or “no match” or “in valid mass”, were also filtered out. Finally, filtered compounds were manually validated by matching fragmentation spectra to those of available standards or to spectra reported in the literature.

1. **Shotgun LC-MS proteomics**

The peptide mixture was dissolved in 20 µL buffer A (2% acetonitrile: 98% water with 0.1% formic acid) and separated by Dionex Ultimate 3000 HPLC System connected to a Thermo Orbitrap Fusion mass spectrometer equipped with an online Nanospray Flex™ Ion Source. Four μL peptide sample was loaded onto Thermo Trap Cartridge (PepMap™ Neo, C18, 100 Å, 300 μm x 5 mm) and eluted from analytical column (Acclaim PepMap C18, 75 μm x 25 cm) by using a 120 min linear gradient from 5% to 30% of solvent B (90% acetonitrile: 10% water with 0.1% formic acid), followed by column wash and calibration at a flow rate of 300 nL/min. The Orbitrap Fusion mass spectrometer was operated in the data-dependent acquisition (DDA) mode. The MS1 survey scan of parent ion was from 350–1500 m/z, and data was acquired at a high resolution of 120,000 (m/z 200), the AGC target was set to 3 × 105 and the maximum injection time was 100 ms. The second stage of mass spectrometry (MS2) scans was performed on IonTrap at rapid scan rate, with dynamic exclusion, 50 seconds; cycle time, 3 seconds; isolation width, 0.7 m/z and MIPS mode as peptide. Ions with charge states 2-7 were sequentially fragmented by collision induced dissociation (CID) with a fixed collision energy of 35%. For the setting of the Nanospray Flex ion source, the spray voltage of 2.3 kV was applied on Thermo Scientific Nano Bore steel emitter and ion transfer tube temperature was set at 275°C.

Protein identifications were performed using the MASCOT search engine (version 2.3.2; Matrix Science, London, UK) embedded into Proteome Discoverer 2.2 (Thermo Fisher Scientific, Massachusetts, USA). The LC-MS/MS raw data were searched against the against Uniprot Human database (version 2022 containing 149,380 protein sequences). Proteome Discoverer parameters were set as follows: MS1 mass tolerance was set to 10ppm while MS2 mass tolerance was 0.6 Da for IonTrap detection, trypsin was chosen as the enzyme, carbamidomethylation of cystine was selected as fixed modification and oxidation of methionine, deamidation of asparagine and glutamine were specified as dynamic modifications. Only proteins with Mascot score ≥21 (p<0.05), at least one unique peptide and that unique peptide was identified with two peptide-spectrum matches (PSMs), were accepted and included in further analysis. The mass spectrometry proteomics data have been deposited to the ProteomeXchange Consortium via the PRIDE 12 partner repository with the dataset identifier PXD040856.
