# Supplementary material for: Using established biorepositories for emerging research questions: a feasibility study
Source: Clin Proteomics. 2024 Aug 17;21:54. doi: 10.1186/s12014-024-09504-6 (PMC11330044; doi:10.1186/s12014-024-09504-6)
Supplement: Supplementary file 2 — Additional file 2. [file 12014_2024_9504_MOESM2_ESM.docx]

Additional File 2 – Supplementary Figures

Applying established serum repositories for emerging research questions: a feasibility study.

**Figure S1.** Bar charts depicting the total protein content in plasma (n=30) and serum samples (n=30).


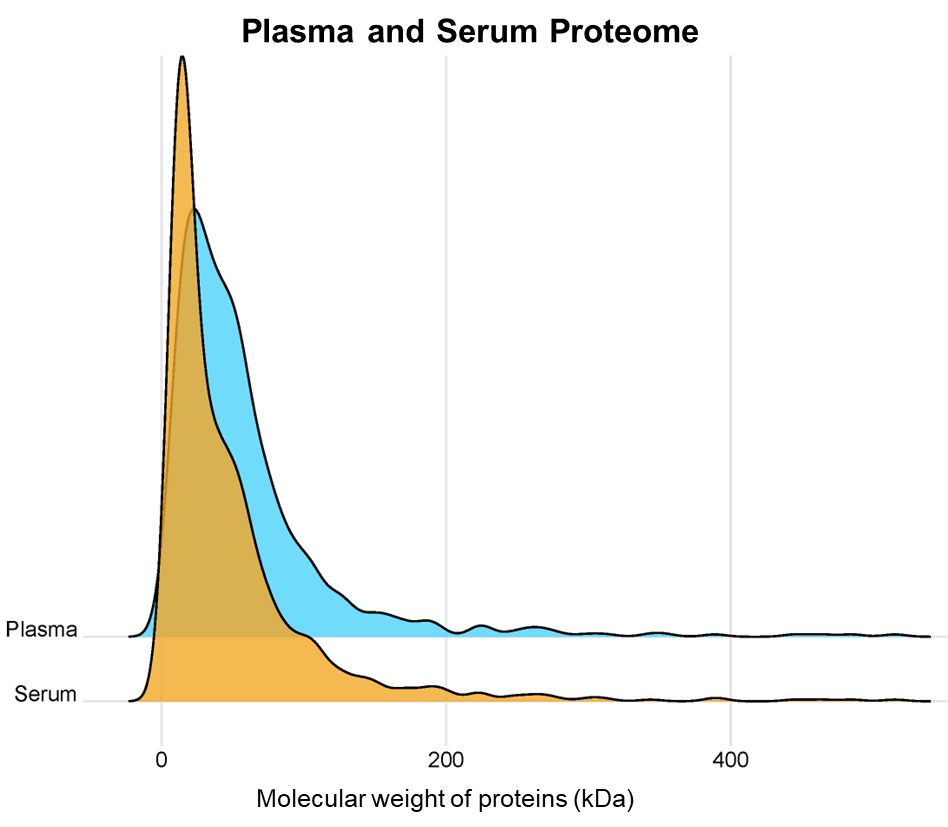


**Figure S2.** Ridgeline plots showing the distributions of the molecular weight of proteins identified in plasma and serum samples.
