# Supplementary material for: Using established biorepositories for emerging research questions: a feasibility study
Source: Clin Proteomics. 2024 Aug 17;21:54. doi: 10.1186/s12014-024-09504-6 (PMC11330044; doi:10.1186/s12014-024-09504-6)
Supplement: Supplementary file 3 — Additional file 3. [file 12014_2024_9504_MOESM3_ESM.docx]

Additional File 3 – Supplementary Tables

Applying established serum repositories for emerging research questions: a feasibility study.

**Table S1.** Demographic characteristics of the kidney recipient serum (PROCARE) and kidney donor plasma (QUOD) study populations. Standard deviations were calculated. PROCARE samples years of collection: 1996-2005, year of analysis: 2023. QUOD samples years of collection: 2013-2018, year of analysis: 2021. N=30 for both groups.

| **Clinical Characteristics** | | **Plasma (n=30)** | **Serum (n=30)** |
| --- | --- | --- | --- |
| Patient age (years, average ± SD) | | 63.6±8.0 | 50.1±11.9 |
| Patient sex | Male (%) | 63.3 | 33.3 |
|  | Female (%) | 36.7 | 66.7 |
| Patient BMI | | 26.2±4.8 | 24.7±3.9 |
| Sample age (years, average ± SD) | | 4.6±1.1 | 20.1±2.9 |
